# DNA preserved in jetsam whale ambergris

**DOI:** 10.1098/rsbl.2019.0819

**Published:** 2020-02-05

**Authors:** Ruairidh Macleod, Mikkel-Holger S. Sinding, Morten Tange Olsen, Matthew J. Collins, Steven J. Rowland

**Affiliations:** 1 Section for EvoGenomics, The GLOBE Institute, University of Copenhagen, Øster Farimagsgade 5, 1353 København K, Denmark; 2 Homerton College, University of Cambridge, Hills Road, Cambridge CB2 8PH, UK; 3 Molecular Population Genetics, Smurfit Institute of Genetics, Trinity College Dublin, Dublin 2, Ireland; 4 McDonald Institute for Archaeological Research, Department of Archaeology and Anthropology, University of Cambridge, West Tower, Downing Street, Cambridge CB2 3ER, UK; 5 Biogeochemistry Research Centre, University of Plymouth, Drake Circus, Plymouth PL4 8AA, UK

**Keywords:** ambergris, ancient DNA, sperm whale, coprolith, shotgun sequencing

## Abstract

Jetsam ambergris, found on beaches worldwide, has always been assumed to originate as a natural product of sperm whales (Physeteroidea). However, only indirect evidence has ever been produced for this, such as the presence of whale prey remains in ambergris. Here, we extracted and analysed DNA sequences from jetsam ambergris from beaches in New Zealand and Sri Lanka, and sequences from ambergris of a sperm whale beached in The Netherlands. The lipid-rich composition of ambergris facilitated high preservation-quality of endogenous DNA, upon which we performed shotgun Illumina sequencing. Alignment of mitochondrial and nuclear genome sequences with open-access reference data for multiple whale species confirms that all three jetsam samples derived originally from sperm whales (*Physeter macrocephalus*). Shotgun sequencing here also provides implications for metagenomic insights into ambergris-preserved DNA. These results demonstrate significant implications for elucidating the origins of jetsam ambergris as a prized natural product, and also for the understanding of sperm whale metabolism and diet, and the ecological mechanisms underlying these coproliths.

## 1. Introduction

Ambergris, a known natural product of the sperm whale [1–4], is also found as jetsam on beaches worldwide [5], and has been highly prized for its utility in the perfume industry [6]. Although it has long been held that the jetsam ambergris collected on beaches originates from sperm whales [4], little or no evidence for this has ever been published, and distinctions exist between such samples and samples of ambergris directly taken from sperm whales. For example, jetsam ambergris samples generally contain much higher proportions of the triterpenoid alcohol ambrein and much lower proportions of sterols than do samples of ambergris from sperm whales [5,7]. Conversely though, jetsam ambergris sometimes does contain fragments of squid beaks [4], and since cephalopods, such as squid, constitute the major dietary component of sperm whales, this has been cited as evidence of an origin of the jetsam coproliths from sperm whales. It is even theorized ambergris may originate as a pathological secretion from the irritant of the hard squid beak chitin [8]. However, other marine mammal species (e.g. members of Globicephala and Ziphiidae) also predate on squid [9–11], and some (including dwarf and pygmy sperm whales) are also cited as potential sources of ambergris [4]. Therefore, to further elucidate the origin of jetsam ambergris, we analysed DNA from an ambergris sample collected from a sperm whale beached in The Netherlands and compared it with DNA sequences isolated from jetsam ambergris collected from beaches in New Zealand and Sri Lanka.

Ambergris is held to be predominantly composed of ambrein due to its production from squalene, a common metabolic product in many organisms [12]. This process may be induced by gut microbial influence, and precipitates in dense, solid masses within the whale colon [7]. The coprolitic accretions that result are compositionally well-suited to preserving DNA from the colon since ambrein is hydrophobic and apparently resistant to degradation within the acidic enteric environment. Evidence from radiocarbon dating certainly indicates resistance to microbial and photo-degradation in the marine environment for up to a millennium in some jetsam ambergris samples [13]. We hypothesized that such material might provide an opportune cache for preserving DNA, even after prolonged exposure to detrimental conditions at sea.

## 2. Material and methods

Jetsam ambergris specimens from the North Sea, the Indian Ocean and the Pacific were analysed, representing the material's global distribution [13]. Three jetsam ambergris specimens (one from Sri Lanka, two from Pitt Island, New Zealand) were subsampled for DNA extraction. A fourth specimen originated from dissection of a male sperm whale beached in December 2012, at Razende Bol near Texel, The Netherlands. The latter ‘fresh’ ambergris, from a confirmed sperm whale carcass, provided a known comparison to the jetsam specimens with unconfirmed biological history. Specimens of ambergris were obtained and analysed for ambrein and faecal sterol content in previous studies [5,7].

**Figure 1.**
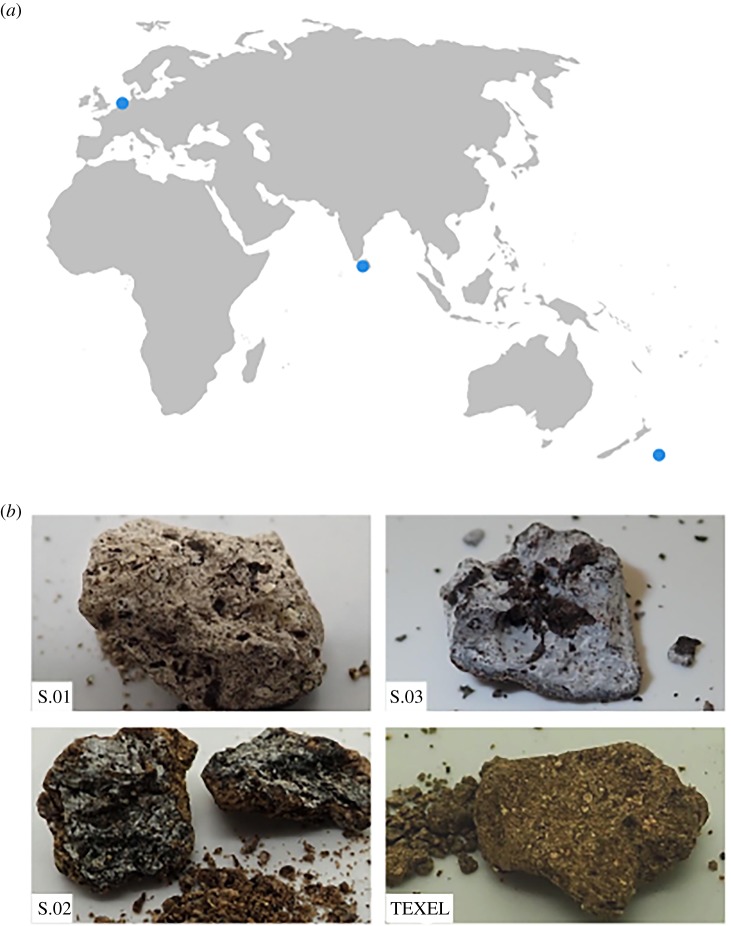
Details for ambergris samples analysed. (*a*) Map showing localities where ambergris samples were originally found. (*b*) Photographs showing high diversity in physical characteristics of ambergris fragments: TEXEL151212 (from dissected whale specimen) was grainy in consistency, while jetsam samples superficially appeared more dense and heterogeneous, and were internally equigranular and significantly paler in colour.

DNA extraction and sequencing were undertaken at the GLOBE Institute, University of Copenhagen, in a dedicated ancient DNA laboratory following strict procedures for minimization of contamination. Approximately 120 mg was subsampled (figure 1 and table 1) for DNA extraction. Samples were incubated in 400 µl proteinase K-containing buffer following Gilbert *et al.* [14] at 56°C for 10 h; supernatants were then treated using a phenol–chloroform step following Carøe *et al*. [15] and purified using Monarch DNA Cleanup Columns (5 µg) (New England Biolabs, Beverly, MA, USA) according to the manufacturer's guidelines. Double-stranded libraries were built from DNA extracts following the BEST protocol [15], designed and proven specifically for sequencing of ancient and degraded DNA. Libraries were amplified and indexed through PCR using PfuTurbo Cx Hotstart (Agilent Technologies, Santa Clara, CA, USA) according to the manufacturer's guidelines. Products were pooled at equimolar concentration before sequencing on an Illumina HiSeq 4000 (Illumina, San Diego, CA, USA) platform, using 80 bp single end read chemistry at the Danish National High-throughput DNA Sequencing Centre, Copenhagen, Denmark.

**Table 1.** Details of sample find localities, masses of original coproliths, subsampled masses used for DNA extraction, and percentage ambrein component (based on DCM-soluble fraction [7,13]).

sample	location	total mass (g)	analysed mass (mg)	% ambrein
S.01	Pitt Island, New Zealand	50	96	92
S.02		20	110	83
S.03	west Sri Lanka	101	188	60
TEXEL151212	Texel, Netherlands	83000	92	93

**Table 2.** Results from sequencing and sequence alignment for *P. macrocephalus* mitochondrial and whole genome references. Coverage estimations are calculated from unique reads aligned with reference sequences. Despite low coverage for S.01, sufficient alignment data exist for species attribution to *P. macrocephalus*, confirmed by phylogenetic model below.

sample	total retained reads	average retained read length (bp)	total aligned reads (mtDNA)	times of coverage (mtDNA)	total aligned reads (whole genome)	times of coverage (whole genome)
S.01	77 261 083	72.9	43	0.175	12 782	0.0000546
S.02	89 486 411	71.7	2440	1.648	26 169	0.000135
S.03	71 907 406	68.3	40 235	9.717	2 447 082	0.00426
TEXEL151212	92 385 587	62.6	71 190	19.654	3 099 642	0.00639

Sequence analysis was undertaken on the high-performance computing facility at the University of Copenhagen, with FASTQ files processed using the PALEOMIX pipeline (v. 1.2.13) [16]. FastQC v. 0.11.8 [17] was initially used for quality control of raw sequence data. Adapters were trimmed using AdapterRemoval v. 2.3.1 [18], with reads less than 25 bp also removed. Reads were then mapped to reference sequences using BWA [19], also applying mapDamage2.0 [20] for basic degradation quantification, producing alignments with references sequences. ANGSD [21] was then used to produce sequences in FASTA format.

Uncertainty around the origin and the biological mechanisms for the production of ambergris prompted us to consider multiple possible candidate cetacean and pinniped species in sequence analysis. Species identity was inferred by mapping success and phylogenetic relationship to 19 cetacean and pinniped candidate species in NCBI RefSeq (see the electronic supplementary material). These species were selected based on potential suitability as deep-diving marine mammals filling a similar ecological niche to sperm whales, to rule out such species being co-adapted to produce ambergris. Sample sequences were concatenated and aligned using MAFFT v. 7.392 [22]. Phylogenetic tree models were then produced in MEGA X [23] using the maximum-likelihood method with the Hasegawa–Kishino–Yano model [24], with distances estimated by the maximum composite likelihood approach (details of all reference sequences used are included in the electronic supplementary material).

## 3. Results

**Figure 2.**
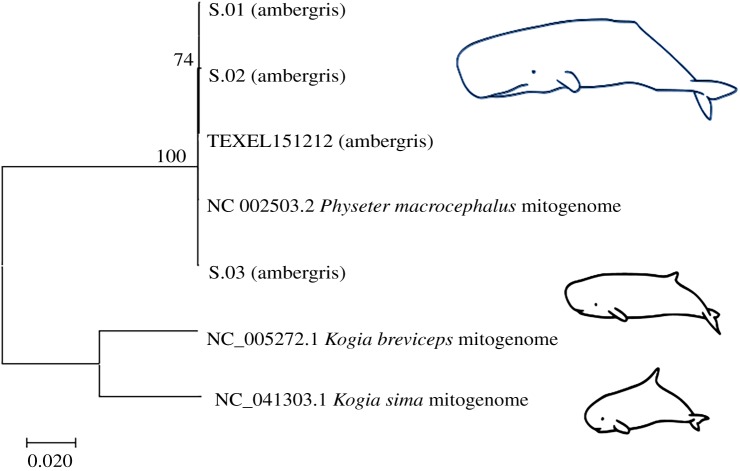
Maximum-likelihood phylogenetic tree model generated from reference sequences and aligned sample mitochondrial genomes. Samples are clearly situated as grouping with sperm whale (*P. macrocephalus*) rather than dwarf and pygmy sperm whales (*Kogia* spp.). This tree reflects the highest log-likelihood model, values reflect the percentage of trees computed in which the associated taxa were clustered, indicating confidence in positioning, and branch lengths measure the number of substitutions at each site (see scale). Figure produced in MEGA X [23]. Whale depictions from: https://commons.wikimedia.org/wiki/File:Sperm whales_size.svg. A phylogenetic tree including all 19 candidate species is presented in electronic supplementary material, figure S1.

The phylogenetic analyses unequivocally supported the sperm whale origin of the four ambergris samples (figure 2; electronic supplementary material, figure S1). Likewise, alignment with the *Physeter macrocephalus* mitochondrial reference genome from NCBI (NC_002503.2) produced the highest coverage results for all samples of all the alignments made and provides a confident attribution, though with significant variations in success between samples. Sequencing of the sample from a stranded sperm whale (TEXEL151212) produced by far the highest coverage (approx. 20×) for sperm whale mitochondrion, while one of the jetsam samples from Pitt Island (S.01) only yielded approximately 0.2× coverage (see table 2). Alignments with *Kogia sima* (dwarf sperm whale) and *Kogia breviceps* (pygmy sperm whale) reference mitochondrial genomes (NC_041303.1, NC_005272.1) also yielded coverage (details in electronic supplementary material), though many highly conserved functional regions are shared between analysed species, resulting in high sequence similarity [25]. However, coverage for kogiid species was typically around a factor of 10 less than for *Physeter*. Alignment with the *P. macrocephalus* whole nuclear reference genome (ASM283717v2) was also successful, though this is more apparent in comparisons of total number of reads mapped to the genome. Alignment for *Architeuthis dux* (giant squid), a reputed common prey of sperm whales (e.g. [26]), was unsuccessful, but this is not their predominant prey [26].

Results from MapDamage show remarkably little inter-sample variation affecting C to T transitions at the 5′ strand ends, though a higher percentage of G to A transitions at the 3′ end exists for S.01 (details in electronic supplementary material, figure S2), indicating possible higher biomolecular degradation. Generally, however, very little chemical modification has occurred, and the distribution of alterations across strands remains uniform and flat.

## 4. Discussion

This study has demonstrated that three jetsam ambergris samples can confidently be attributed to sperm whale through DNA extraction. While confirmation of a sperm whale origin for jetsam ambergris is not a surprising result, the present study is the first in providing a significant proof-of-concept in retrieving endogenous DNA from ambergris and successfully using it for organism identification. Importantly, the origin of all three jetsam ambergris samples studied here can confidently be identified as sperm whale on the basis of not only genetic alignment success, but also modelling of mitochondrial genomes in phylogenetic relatedness trees, including for a large sample of outgroup marine mammal taxa. However, although all samples analysed here were identified as originating from sperm whale, it is still quite possible that other closely related deep-diving marine mammals (such as the dwarf and pygmy sperm whales) might produce ambergris [4] and have simply still not been recorded as doing so to date.

The predominant cause of the dramatic variation in genetic coverage seen between samples is unclear. Analysis of DNA degradation in mapDamage2.0 shows little correlation with alignment coverages, as might be expected, and there is also little variation between ambrein content in samples that might be expected to contribute to differential DNA preservation. The precise age of the present jetsam samples is unknown, although previous studies have successfully radiocarbon dated other ambergris samples [13]. However, radiocarbon dating of relatively recent samples is problematical owing to the impact of fossil fuel emissions [27], and radiocarbon dates since the increase of anthropic carbon release are unreliable. Producing a consistent degradation rate for G to A transitions in reliably dated older samples might, in future, aid a better understanding of differential DNA damage. Another option for future research might be studies of glutamine deamidation and aspartic acid racemization from analysis of organic peptides possibly also present in ambergris [28,29]. Alternatively, however, intra-sample variation in DNA and ambrein concentration might just as likely account for low coverage in sample S.01, while more recent exposure to sperm whale tissue undoubtedly accounts for the high coverage in beached whale sample TEXEL151212.

The preservational potential of ambrein precipitates for DNA extends not just to endogenous whale genetics, but also to metagenomic coverage of the whale gut microbiome, and potentially also the DNA of their prey. For example, DNA may also remain within partially or undigested squid beaks found in sperm whale faeces [30], and in ambergris [31], which are even theorized to be a pathological cause of ambergris secretion [8]. Understanding of the prokaryotic composition of the microbiome environment in ambergris could also further elucidate the origin of ambergris, particularly in the conversion of squalene to ambrein and the process by which ambergris appears to form in layers of accretion. Further analyses on endogenous DNA retrieval from jetsam ambergris, including also DNA from whale gut microbiota and prey, would yield significantly greater insights into sperm whale ecology, evolution and metabolism.

## 5. Conclusion

Jetsam ambergris has long been an enigmatic material, subject to discussion and analyses in scientific publications since the eighteenth century [1,31]. This study is the first to our knowledge to present final confirmation of the biological origin of jetsam ambergris samples as sperm whales, through DNA analysis. Beyond this, however, the present study lays out the potential of ambergris as a new source of genetic data related to sperm whales with a considerable longevity across time. Greater elucidation remains to be achieved through the study of the preservational conditions of DNA in ambrein and of the differential effects from multiple factors. However, the potential implications for aiding our understanding of past population dynamics in whales and their ecologically associated taxa may be profound. The oldest-known ambergris found within Pleistocene deposits feature permineralized squid beaks containing amino acids endogenous to squid, which the authors attribute to the preservational capacity of the local sediment [32,33]. Although it is unlikely DNA will be preserved for such an age (1.75 Ma), this finding might also be attributable to the effectiveness of ambergris and ambrein as preservational substrates. A great deal is still unknown about the ecology and adaptation of the marine giants formerly characterized as semi-mythical beasts, and ambergris may now prove a small but significant key to understanding some further aspects of them.

## Supplementary Material

DNA Preserved in Jetsam Whale Ambergris: Supplementary Information

## Data accessibility

In support of data open-access, all data generated in this study has been made available on the Zenodo data repository [34]: https://doi.org/10.5281/zenodo.3528073.
