# Supplementary material for: DNA preserved in jetsam whale ambergris
Source: Biol Lett. 2020 Feb 5;16(2):20190819. doi: 10.1098/rsbl.2019.0819 (PMC7058951; doi:10.1098/rsbl.2019.0819)

**DNA Preserved in Jetsam Whale Ambergris : Supplementary Information**

Ruairidh Macleod^1,2*^, Mikkel-Holger S. Sinding^1,3^, Morten Tange Olsen^1^, Matthew J. Collins^1,4^, and Steven J. Rowland^5^

^1^Section for EvoGenomics, The GLOBE Institute, Øster Farimagsgade 5, University of Copenhagen, 1353 København K, Denmark

^2^Homerton College, University of Cambridge, Hills Road, Cambridge, CB2 8PH, UK.

^3^Molecular Population Genetics, Smurfit Institute of Genetics, Trinity College Dublin, Dublin 2, Ireland.

^4^McDonald Institute for Archaeological Research, Department of Archaeology and Anthropology, West Tower, Downing St, University of Cambridge, Cambridge CB2 3ER, UK.

^5^Biogeochemistry Research Centre, University of Plymouth, Drake Circus, Plymouth, PL4 8AA, UK.

*****Correspondence to: [ram88@cam.ac.uk](mailto:ram88@cam.ac.uk) (R.M.)

#### Sample sequence data for alignments with *P. macrocephalus* whole genome ASM283717v2 (NCBI) and mitochondrial genome (NCBI Reference Sequence: NC_002503.2) is available in FASTA format in the open data repository Zenodo: **doi.org/10.5281/zenodo.3528073**

**Table S1.** Further sample details: results of mass spectral analyses and radiocarbon dating where attempted.

| **Sample ID** | **Beach Location** | **Country** | **%DCM extractable** | **% ambrien** | **δ13C ‰** | **14C age (y)** | **F14C** | **Cal. age** |
| --- | --- | --- | --- | --- | --- | --- | --- | --- |
| S.01 | Pitt Island | New Zealand | 100 | 92 | n.d. | n.d. | n.d. | n.d. |
| S.02 | Pitt Island | New Zealand | 96 | 83 | n.d. | n.d. | n.d. | n.d. |
| S.03 | Sri Lanka | Sri Lanka | 100 | 60 | n.d. | n.d. | n.d. | n.d. |
| TEXEL151212 | Razende Bol, near Texel | The Netherlands | 93 | 93 | -21.1 | -486 | 1.062±0.003 | Modern |

**Table S2.** Screening results for ambergris extracted DNA sequences generated using the PALEOMIX pipeline (v1.2.13) and ANGSD. Analysis refers to reads mapped to the sperm whale whole reference genome, and deamination rates at 5’ ends of DNA strands were produced from MapDamage2.0.

| **Sample ID** | **Sampled mass (mg)** | **Raw reads** | **Retained reads** | **Fraction reads discarded (%)** | **Average retained read length (bp)** | **C-T at 5’ (%)** |
| --- | --- | --- | --- | --- | --- | --- |
| S.01 | 96 | 77672509 | 77261083 | 0.53 | 72.909 | 1.097 |
| S.02 | 110 | 89866772 | 89486411 | 0.42 | 71.655 | 0.673 |
| S.03 | 188 | 72019240 | 71907406 | 0.16 | 68.278 | 0.531 |
| TEXEL151212 | 92 | 92793431 | 92385587 | 0.44 | 68.620 | 0.135 |

**Table S3.** Coverage results for sequence alignments with multiple reference taxa mitochondrial genomes.

| **Sample** | **X Coverage**  **(*P. macrocephalus* mitchondrion)** | **X Coverage**  **(*P. macrocephalus* whole genome)** | **Coverage**  **(*K. sima* mitchondrion)** | **Coverage**  **(*K. breviceps* mitchondrion)** | **Coverage**  **(*A. dux* mitchondrion)** |
| --- | --- | --- | --- | --- | --- |
| S.01 | 0.1753104456 | 0.0000546412565633 | 0.0196305553862 | 0.0122516152627 | 0 |
| S.02 | 1.648039932 | 0.000134949776367 | 0.101200999817 | 0.12696574424 | 0 |
| S.03 | 9.716946676 | 0.00426244115556 | 0.635920258489 | 0.770815555285 | 0 |
| TEXEL151512 | 19.65418797 | 0.0063855660763 | 1.39645186856 | 1.48518834573 | 0 |

**Table S4.** Details of reference genome sequences used for analyses. Comparison species were selected on the basis of known dietary behaviour: squid predation and deep-diving.

| **Species** | **Common Name** | **Genome type** | **NCBI reference ID** | **Publication Reference/Authors** |
| --- | --- | --- | --- | --- |
| *Physeter macrocephalus* | sperm whale | Mitochondrion | NCBI Reference Sequence: NC_002503.2 | Arnason U, Gullberg A, Gretarsdottir S, Ursing B, Janke A. The mitochondrial genome of the sperm whale and a new molecular reference for estimating eutherian divergence dates. Journal of Molecular Evolution. 2000 Jun 1;50(6):569-78. |
| *Physeter macrocephalus* | sperm whale | Whole (shotgun sequencing) | Genome Assembly: ASM283717v2 | Arnason U, Gullberg A, Gretarsdottir S, Ursing B, Janke A. The mitochondrial genome of the sperm whale and a new molecular reference for estimating eutherian divergence dates. Journal of Molecular Evolution. 2000 Jun 1;50(6):569-78. |
| *Kogia breviceps* | pygmy sperm whale | Mitochondrion | GenBank: AJ554055.1 | Arnason U, Gullberg A, Janke A. Mitogenomic analyses provide new insights into cetacean origin and evolution. Gene. 2004 May 26;333:27-34. |
| *Kogia sima* | dwarf sperm whale | Mitochondrion | NCBI Reference Sequence: NC_041303.1 | Shan L, Tian R, Liu Y. The complete mitochondrial genome of the dwarf sperm whale Kogia sima (Cetacea: Kogiidae). Mitochondrial DNA Part B. 2019 Jan 2;4(1):72-3. |
| *Mesoplodon mirus* | True's beaked whale | Mitochondrion | NCBI Reference Sequence: NC_042217.1 | Einfeldt AL, Paterson IG, Feyrer LJ. Complete mitochondrial genomes and phylogeny of two rare whale species: True’s (Mesoplodon mirus: True 1913) and Sowerby’s (Mesoplodon bidens: Sowerby 1804) beaked whales. Mitochondrial DNA Part B. 2019 Jan 2;4(1):275-6. |
| *Mesoplodon stejnegeri* | Stjeneger's beaked whale | Mitochondrion | NCBI Reference Sequence: NC_036997.1 | Lee K, Lee J, Cho Y, Kim HW, Park KJ, Sohn H, Choi YM, Kim HK, Jeong DG, Kim JH. Characterization of the complete mitochondrial genome of Stejneger’s beaked whale, Mesoplodon stejnegeri (Cetacea: Ziphiidae). Conservation genetics resources. 2018 Dec 1;10(4):839-42. |
| *Indopacetus pacificus* | Longman's beaked whale | Mitochondrion | NCBI Reference Sequence: NC_034348.1 | Yao, C.-J., Audira, G. and Hsiao, C.-D. The complete mitogenomes of Indo-pacific Beaked Whale (Indopacetus pacificus) (Chordata: Ziphiidae): an endangered species which is considered to be the world's rarest cetacean (unpublished) |
| *Mesoplodon ginkgodens* | Gingko-toothed beaked whale | Mitochondrion | NCBI Reference Sequence: NC_027593.1 | Yao CJ, Chen CH, Hsiao CD. The complete mitogenome of Ginkgo-toothed beaked whale (Mesoplodon ginkgodens)(Chordata: Ziphiidae). Mitochondrial DNA Part A. 2016 Jul 3;27(4):2846-7. |
| *Mesoplodon grayi* | Gray's beaked whale | Mitochondrion | NCBI Reference Sequence: NC_023830.1 | Thompson, K.F., Patel, S., Williams, L., Tsai, P., Baker, C.S., Constantine, R. and Millar, C.D. High coverage of the complete mitochondrial genome of the Gray's beaked whale, Mesoplodon grayi, using Illumina next generation sequencing (unpublished) |
| *Mesoplodon densirostris* | Blainville's beaked whale | Mitochondrion | NCBI Reference Sequence: NC_021974.2 | Morin, P.A., Duchene, S., Lee, N., Durban, J. and Claridge, D. (unpublished) |
| *Mesoplodon europaeus* | Gervais' beaked whale | Mitochondrion | NCBI Reference Sequence: NC_021434.2 | Morin, P.A., Duchene, S., Lee, N., Durban, J. and Claridge, D. (unpublished) |
| *Berardius bairdii* | Baird's beaked whale | Mitochondrion | NCBI Reference Sequence: NC_005274.1 | Arnason U, Gullberg A, Janke A. Mitogenomic analyses provide new insights into cetacean origin and evolution. Gene. 2004 May 26;333:27-34. |
| *Hyperoodon ampullatus* | Northern bottlenose whale | Mitochondrion | NCBI Reference Sequence: NC_005273.1 | Arnason U, Gullberg A, Janke A. Mitogenomic analyses provide new insights into cetacean origin and evolution. Gene. 2004 May 26;333:27-34. |
| *Ziphius cavirostris* | Cuvier's beaked whale | Mitochondrion | NCBI Reference Sequence: NC_021435.1 | Morin PA, Duchene S, Lee N, Durban J, Claridge D. Preliminary analysis of mitochondrial genome phylogeography of Blainville’s, Cuvier’s and Gervais’ beaked whales. International Whaling Commission, Scientific Meeting 64, Panama City, Panama. SC/64/SM14; 2012. |
| *Mesoplodon bidens* | Sowerby's beaked whale | Mitochondrion | NCBI Reference Sequence: NC_042218.1 | Einfeldt AL, Paterson IG, Feyrer LJ. Complete mitochondrial genomes and phylogeny of two rare whale species: True’s (Mesoplodon mirus: True 1913) and Sowerby’s (Mesoplodon bidens: Sowerby 1804) beaked whales. Mitochondrial DNA Part B. 2019 Jan 2;4(1):275-6. |
| *Globicephala melas* | long-finned pilot whale | Mitochondrion | NCBI Reference Sequence: NC_019441.1 | Vilstrup JT, Ho SY, Foote AD, Morin PA, Kreb D, Krützen M, Parra GJ, Robertson KM, de Stephanis R, Verborgh P, Willerslev E. Mitogenomic phylogenetic analyses of the Delphinidae with an emphasis on the Globicephalinae. BMC evolutionary biology. 2011 Dec;11(1):65. |
| *Globicephala macrorhynchus* | short-finned pilot whale | Mitochondrion | NCBI Reference Sequence: NC_019578.2 | Morin PA, Archer FI, Foote AD, Vilstrup J, Allen EE, Wade P, Durban J, Parsons K, Pitman R, Li L, Bouffard P. Complete mitochondrial genome phylogeographic analysis of killer whales (Orcinus orca) indicates multiple species. Genome research. 2010 Jul 1;20(7):908-16. |
| *Mirounga leonina* | southern elephant seal | Mitochondrion | NCBI Reference Sequence: NC_008422.1 | Arnason U, Gullberg A, Janke A, Kullberg M, Lehman N, Petrov EA, Väinölä R. Pinniped phylogeny and a new hypothesis for their origin and dispersal. Molecular phylogenetics and evolution. 2006 Nov 1;41(2):345-54. |
| *Pseudorca crassidens* | False killer whale | Mitochondrion | GenBank: HM060332.1 | Morin PA, Archer FI, Foote AD, Vilstrup J, Allen EE, Wade P, Durban J, Parsons K, Pitman R, Li L, Bouffard P. Complete mitochondrial genome phylogeographic analysis of killer whales (Orcinus orca) indicates multiple species. Genome research. 2010 Jul 1;20(7):908-16. |
| *Feresa attenuata* | Pygmy killer whale | Mitochondrion | NCBI Reference Sequence: NC_019588.1 | Vilstrup JT, Ho SY, Foote AD, Morin PA, Kreb D, Krützen M, Parra GJ, Robertson KM, de Stephanis R, Verborgh P, Willerslev E. Mitogenomic phylogenetic analyses of the Delphinidae with an emphasis on the Globicephalinae. BMC evolutionary biology. 2011 Dec;11(1):65. |

**Figure S1.** Full maximum likelihood phylogenetic tree model of aligned sample sequences (with P. macrocephalus mitochondrian) and reference mitochondrial genomes of all considered marine mammal species.This tree reflects the highest log likelihood model, and values reflect the percentage of trees computed in which the associated taxa were clustered, indicating confidence in positioning, and branch lengths measure the number of substitutions at each site (see scale). Figure produced in MEGAX (Kumar et al. 2018).


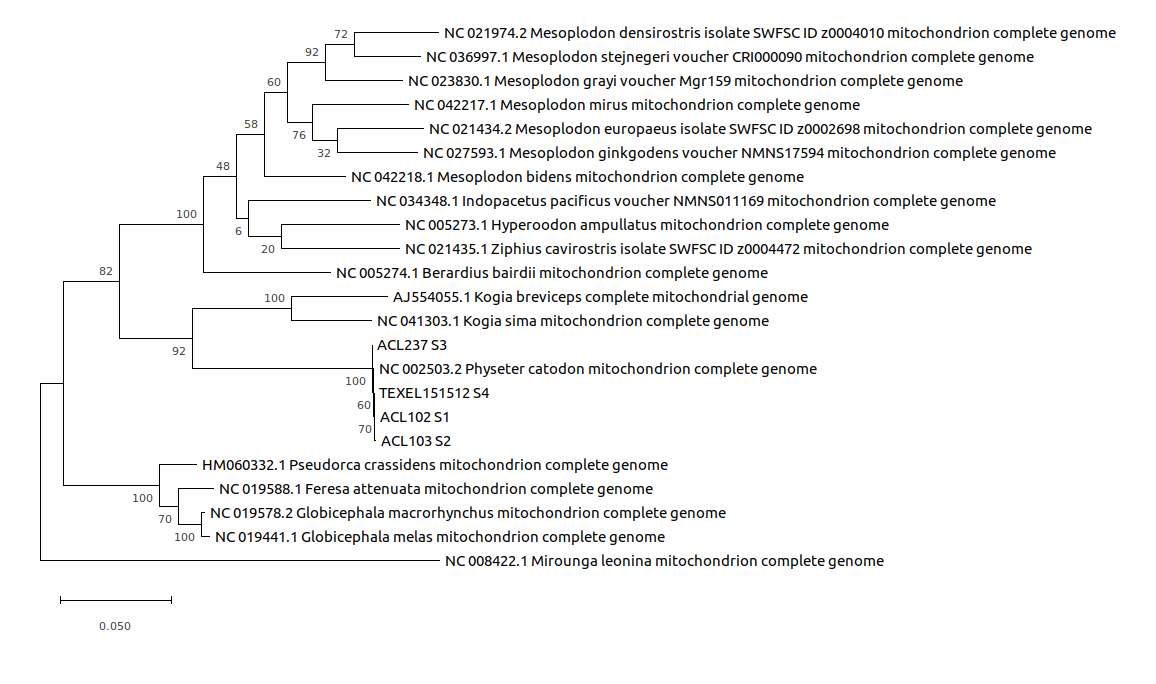


**Figure S2.** MapDamage2.0 outputs for ambergris sequences aligned with *P. macrocephalus* whole reference genome. The apparently more dramatic peaks present in S.01 are an outcome of significantly fewer reads being successfully mapped to the reference genome in this example. The four smaller plots for each sample display base frequency within and outside reads (grey brackets correspond to read), while lower plots show positions’ specific substitutions at 5’ (left) and 3’ (right) strand ends.
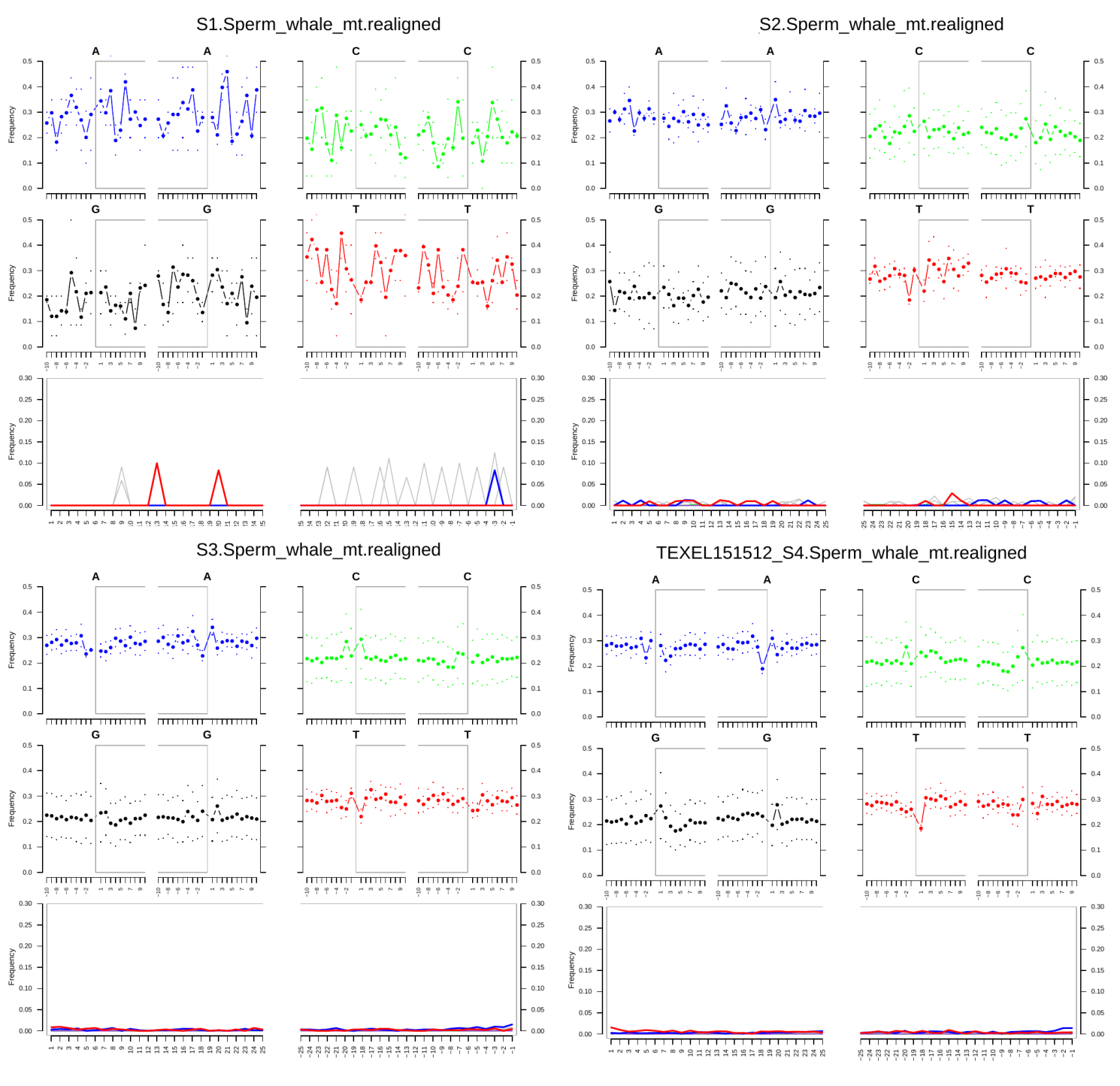

Supplement: DNA Preserved in Jetsam Whale Ambergris: Supplementary Information [file rsbl20190819supp1.docx]
